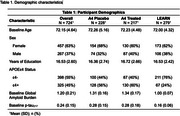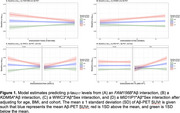# X‐linked gene expression is associated with plasma *p*‐tau217 levels via Aβ‐PET and sex in cognitively unimpaired older adults

**DOI:** 10.1002/alz70855_101138

**Published:** 2025-12-23

**Authors:** Mabel Seto, Hannah M Klinger, Vaibhav A Janve, Jane A Brown, Colin Birkenbihl, Gillian T Coughlan, Diana L Townsend, Ting‐Chen Wang, Michael J. Properzi, Michelle J. Clifton, Bernard J Hanseeuw, Jasmeer P. Chhatwal, Hyun‐Sik Yang, Robert A. Rissman, Paul S. Aisen, Madison Cuppels, Michael C. Donohue, Rema Raman, Keith A. Johnson, Reisa A. Sperling, Logan Dumitrescu, Timothy J. Hohman, Rachel F. Buckley

**Affiliations:** ^1^ Massachusetts General Hospital, Harvard Medical School, Boston, MA, USA; ^2^ Vanderbilt Memory and Alzheimer's Center, Vanderbilt University Medical Center, Nashville, TN, USA; ^3^ Vanderbilt Genetics Institute, Vanderbilt University Medical Center, Nashville, TN, USA; ^4^ Vanderbilt Memory & Alzheimer's Center, Vanderbilt University Medical Center, Nashville, TN, USA; ^5^ Department of Neurology, Brigham and Women's Hospital, Boston, MA, USA; ^6^ Alzheimer's Therapeutic Research Institute, Keck School of Medicine, University of Southern California, San Diego, CA, USA; ^7^ Alzheimer's Therapeutic Research Institute, University of Southern California, San Diego, CA, USA; ^8^ Brigham and Women's Hospital, Boston, MA, USA; ^9^ Vanderbilt Memory and Alzheimer's Center, Vanderbilt University School of Medicine, Nashville, TN, USA

## Abstract

**Background:**

Higher levels of plasma *p*‐tau_217_ are closely associated with increased Aβ‐PET burden, and subsequent cognitive decline in older adults, supporting it as a sensitive, early marker of AD. Previous studies implicated X‐linked gene expression in AD but were limited to gene expression from postmortem tissue resulting in findings less clinically relevant to early stages of disease. To better inform the relationship between X‐linked genes and AD during the earliest disease processes, we aimed to identify associations between whole blood X‐linked gene expression and plasma *p*‐tau_217_ levels in clinically normal older adults from A4/LEARN.

**Method:**

We leveraged Aβ‐PET(^18^F‐Florbetapir), plasma *p*‐tau_217_(immunoassay, Eli Lilly), and whole blood RNAseq data from 724 cognitively unimpaired participants (72.2years(±4.6); 63%Female; 35%*APOE*ε4+; 26%Aβ+) from the A4 clinical trial at baseline (placebo[31%], treatment[30%]) and the LEARN[39%] observational study. We ran linear regressions adjusting for age, BMI, and cohort to determine associations between the following terms and *p*‐tau_217_(pg/mL): gene, gene*APOEε4, gene*sex, gene*Aβ_continuous_, and gene*sex*Aβ_continuous_. Though focusing on X‐linked genes, results were FDR‐corrected for both autosomal and X‐linked genes (*n* = 20,621).

**Result:**

No X‐linked genes were directly associated with *p*‐tau_217_ levels. 119 X‐linked genes were moderated by Aβ and 27 genes by Aβ*sex on *p*‐tau_217_. Notably, we identified 4 genes previously implicated in AD: *FAM156B, KDM6A, WWC3, and MIDI1IP1*, which are involved in chromatin remodeling, *hippo* pathway signaling, and lipid signaling. In gene*Aβ_continuous_ models, higher *FAM156B* expression (β=‐0.09(0.03), p_FDR_<0.001, Figure 1A) was associated with lower *p*‐tau_217_ levels among individuals with high Aβ‐PET burden whereas higher *KDM6A* expression (β=0.31(0.10), p_FDR_=0.003, Figure 1B) was associated with higher *p*‐tau_217_ levels in both sexes. In females with elevated Aβ‐PET, higher *WWC3* expression was associated with lower *p*‐tau_217_ (β=‐0.44(0.15), p_FDR_=0.004, Figure 1C). In males with high Aβ‐PET burden, both greater *WWC3* (β=0.37(0.14), p_FDR_=0.01, Figure 1C) and *MID1IP1* (β=0.60(0.15), p_FDR_<0.001, Figure 1D) expression was associated with higher *p*‐tau_217_.

**Conclusion:**

Significant whole‐blood X‐linked gene expression associations with *p*‐tau_217_ levels in clinically normal older adults are largely moderated by Aβ‐PET burden and sex. This study identified both protective and risk genes, highlighting novel gene candidates for further validation and supporting the need to study sex chromosomes in AD.